# Development of an HIV-1 Specific Microbicide Using *Caulobacter crescentus* S-Layer Mediated Display of CD4 and MIP1α

**DOI:** 10.1371/journal.pone.0010366

**Published:** 2010-04-28

**Authors:** John F. Nomellini, Carmen Li, Danielle Lavallee, Iryna Shanina, Lisa A. Cavacini, Marc S. Horwitz, John Smit

**Affiliations:** 1 Beth Israel Deaconess Medical Center, Boston, Massachusetts, United States of America; 2 Microbiology and Immunology, The University of British Columbia, Vancouver, British Columbia, Canada; University of Toronto, Canada

## Abstract

The development of alternative strategies to prevent HIV infection is a global public health priority. Initial efforts in anti-HIV microbicide development have met with poor success as the strategies have relied on a non-specific mechanism of action. Here, we report the development of a microbicide aimed at specifically blocking HIV entry by displaying molecular components of the HIV/host cell attachment complex on the surface of *Caulobacter crescentus*, a harmless aquatic bacterium. This bacterium can be readily manipulated to present heterologous proteins at high density on its surface by genetic insertion into its crystalline surface layer protein [1], [2]. In separate constructions, we generated bacteria displaying domain 1 of CD4 and MIP1α. Each moiety reacted with specific antibodies by Western immunoblot and immuno-fluorescence microscopy. Microbicide functionality was assessed using an HIV pseudotype virus assay system representing Clade B subtypes. Bacteria displaying MIP1α reduced infectivity by 35–78% depending on the specific subtype while CD4 display reduced infection by as much as 56%. Combinations of both constructs reduced infectivity by nearly 98%. We demonstrated that HIV infection could be inhibited using a strategy aimed at HIV-specific molecular interactions with *Caulobacter* surface protein display, and that sufficient protein folding and conformation could be mimicked to bind and block entry. Further, this is the first demonstration that *Caulobacter* surface protein display may be a useful approach to preventing HIV infection or other viruses as a microbicide. We propose that this harmless bacterium, which is inexpensive to produce and formulate, might be suitable for topical applications as a viable alternative in the search for effective microbicides to counteract the world wide incidence of HIV infection.

## Introduction

Human Immunodeficiency Virus (HIV) is a growing epidemic and one of the largest global health concerns in this present day. UNAIDS 2008 Report estimated that over 32.9 million people were living with HIV, 2.7 million people became infected in 2007, and there were 2 million reported deaths that year [3]. Due to difficulties in designing an effective HIV vaccine as a consequence of the genetic diversity of the virus, current research has been focusing on the development of microbicides.

In developing countries, women are 3–6 times more likely to become infected with HIV than men due to a lack of female-controlled methods to prevent the transmission of the virus [4]. In women, the main site of HIV entry is the cervicovaginal mucosa. Although the precise cell type and transmission site are not completely understood, it is believed that an appropriately manufactured microbicide will be beneficial as it will offer broad protection against mucosal transmission of HIV at the point of entry. Currently, there are no microbicides on the market but as many as 50 different drugs are currently being tested in clinical trials [5]. Typical methods of delivery include semi-solid aqueous-based gels, vaginal rings, quick-dissolve films, and vaginal tablets [5]. To date, microbicides have shown potential in blocking HIV in tissue culture, but have failed to protect in clinical trials. The primary strategy and likely failing of these early microbicide products is their non-specific mode of action.

Efforts to produce specific microbicide blocking agents using whole organisms have centered on the construction of commensal bacteria, such as Lactobacillus, that would display HIV blocking abilities while colonizing the vaginal mucosa [6], [7], [8]. Inherent in these approaches is the expectation that sufficiently high levels of these bacteria can successfully compete with other microbial flora and thereby be continuously maintained at mucosal surfaces at useful levels for extended periods of time. While competing for space in the microflora population of the vaginal mucosa, these bacteria would also be required to maintain sufficiently high populations as well as levels of secretion and display of agents such as CD4 on the bacteria to effectively block HIV infection.

As a contrast to this approach, we developed a bacterium based microbicide display option that does not rely on the bacterium's ability to compete and survive within the microflora. *Caulobacter crescentus* is not a commensal bacterium; it does not grow at temperatures exceeding ca 32°C and will not grow in the presence of salts at levels typical for sera. But it is capable of display of proteins at very high levels and surface densities. It can be cultivated readily to high densities on defined growth media consisting only of glucose and essential inorganic ions. Although a gram negative organism, its unusual lipopolysaccharide structure appears to have a much reduced sepsis potential, relative to enteric bacteria [9]. With these characteristics we expect the possibility that engineered strains can be formulated to be applied as stabilized killed organisms, designed to be applied to vaginal or other mucosal tissues at relevant times, such prior to sexual acts or childbirth, roughly comparable to spermicide use. This permits maintenance of high levels without the requirement for, or potential negative effects of competition with the existing microflora.

Given the display capabilities of *C. crescentus* we envision the development of a variety of agents expected to interrupt the HIV infection process, and then to apply several simultaneously, to enhance microbicide effects and to minimize the possibility of development of resistance. This includes antibodies directed to HIV gp120, mimics for host receptors and co-receptors for HIV engagement, as well as the ligands for these surface proteins or structural analogues for any of the above. Here we begin with display of domain 1 of CD4, the HIV receptor and MIP1α, the ligand for CCR5, the HIV co-receptor. Considerable evidence exists that demonstrates that binding to block either of these two ligand interactions (CD4:gp120 and MIP1α:CCR5) will inhibit HIV infection [10]. Herein, we demonstrate that separate constructs have the ability to significantly block infection and that the application of both simultaneously has additive microbicide effects.

## Materials and Methods

### Bacterial strains, growth conditions and general plasmid related methods

*Escherichia coli* strain DH5 α (Invitrogen, Carlsbad, CA) was grown at 37°C in Luria Broth (1% tryptone, 0.5% NaCl, 0.5% yeast extract), adding 1.3% agar for plates. The *C. crescentus* strain JS 4022 [11]was propagated in PYE medium (0.2% peptone, 0.1% yeast extract, 0.01% CaCl_2_, 0.02% MgSO_4_), at 30°C. For growth on solid medium, agar was added to 1.3%.

Where necessary, media contained chloramphenicol (CM) at 20 µg/ml (*E. coli*) or 2 µg/ml (*C. crescentus*). Electroporation of *C. crescentus* was performed as previously described [12]. The Nucleic Acid Protein Service unit of the University of British Columbia provided oligonucleotide synthesis and DNA sequencing. Plasmid DNA was isolated using a QIAprep miniprep plasmid isolation kit (QIAGEN), and DNA fragments were recovered from agarose gels using a QIAEX II gel extraction kit (QIAGEN). PCR products were generated using *Pfx* DNA polymerase (Invitrogen, Carlsbad, CA), following the manufacturer's protocol.

### Preparation of *C. crescentus* displaying chimeric S-layer proteins

pT4B (CD4 in pSP65, [13], (obtained from the AIDS Research and Reference Reagent Program, Div. of AIDS, NIAID, NIH: pTB4B from Dr. Richard Axel)) was used as a template and the oligos JN CD4-1 5′GGA AGA TCT ACT AGT GGG GAT ACA GTG GAA CTG ACC3′ and JN CD4-2 5′GGG GCT AGC CTG GTC CTC CAC TTC ACA GAT3′ were used in a PCR reaction to make the DNA fragment that codes for the 81 amino acid CD4 domain 1 segment (GDTVELTCTASQKKSIQFHWKNSNQIKILGNQGSFLTKGPSKLNDRADSRRSLWDQGNFPLIIKNLKIEDSDTYICEVEDQ). Similarly, pCDNA3 CCL3 L1 [14] was used as template and the oligos JN CCR5-1 5′GGA AGA TCT ACT AGT GCA CCA CTT GCT GCT GAC ACG CCG3′  and JN CCR5-2 5′CGG GCT AGC ACT CAG CTC CAG GTC ACT GAC3′ were used to make the DNA fragment that codes for the 70 amino acid MIP1α segment (APLAADTPTACCFSYTSRQIPQNFIADYFETSSQCSKPSVIFLTKRGRQVCADPSEEWVQKYVSDLELSA). The DNA segments produced contained *Bgl*II and *Spe*I restriction sites on the 5′ end and an *Nhe*I site on the 3′ end. The restriction sites arrangement allowed the segments to be directionally cloned into p4ARsaA(723)/GSCC digested with *Bgl*II and *Nhe*I [11].

Following ligation plasmids were introduced into *E. coli* by electroporation. Plasmids were retrieved from selected clones and the inserted sequence confirmed by DNA sequencing before transfer to *C. crescentus* JS4022 by electroporation. The result was the in-frame introduction of the CD4 and MIP1α segments at a site corresponding to amino acid 723 of the native RsaA protein. p4ARsaA(723) (containing only a *Bam*H1 restriction site at the same position) was used as a negative control. Hereafter, the Caulobacter constructs will be referred to as the Cc-CTRL, Cc-CD4 or Cc-MIP1a clones.

Heat-inactivated Caulobacters were prepared by treatment of cultures at 70°C for 4 minutes; killing was confirmed by spread plating.

### Fluorescence microscopy

Antibodies to CD4 (murine monoclonals SIM.2 and SIM.4 and polyclonal sheep anti-human CD4) and MIP1α (polyclonal goat anti-Mip1α) were obtained from the NIH AIDS Reagent and Reference Program. To confirm the presence of displayed proteins on Caulobacter, in a typical experiment 30 µl of cells and 1–5 µl of antibodies were diluted to 200 µl in PYE medium and incubated in ice for 30 min. The mixture was diluted to 1.7 ml with PYE medium and centrifuged at 13,000×g for 4 min. Cell pellets were suspended in 100 µl of PYE and 1 µl anti-mouse, anti-goat or anti-sheep antisera coupled to Alexa488 (Molecular Probes/Invitrogen). After a 20 min on ice the mixture was diluted to 1.7 ml and centrifuged. Pellets were suspended in 20 mM phosphate buffer containing 50% glycerol and 2% n-propyl gallate and examined by epifluorescence microscopy.

### Protein analysis

S-layer and S-layer recombinant proteins were recovered from the cell surface by low-pH extraction method, as described previously [15] Proteins were visualized using sodium dodecyl sulfate-polyacrylamide gel electrophoresis (SDS-PAGE) using 7.5% separating gels and staining with Coomassie brilliant blue R.

### Preparation of Caulobacter cells for binding assay

Caulobacter strain JS4022 with different plasmids were grown in 10 ml PYE medium to an optical density of approximately 1.0 at 600 nm (3.1×10^9^ cells/ml). Cells were centrifuged and suspended in 10 mM potassium phosphate buffer (pH 7.0). This was repeated and cells were re-suspended in 1 ml of the same. Cell density was adjusted to 1×10^10^ cells/ml for binding experiments.

### Plasmid preparation and transfection for virus pseudotype analysis

*E. coli* with plasmids containing the viral DNA cassettes were grown overnight at 37°C in LB broth containing 50 µg/ml of ampicillin. The DNA from the rev/env clones as well as the HIV-env-deficient (SG3Δenv) backbone from the standardized subtype B HIV-1 panel (NIH AIDS Reagent and Reference Program) was then purified using the Sigma GenElute HP Plasmid MaxiPrep Kit. DNA was further purified with two phenol-chloroform extraction steps followed by ethanol precipitation and suspension in 10 mM Tris buffer (pH 7.5) containing 1 mM EDTA. The final concentration of the DNA was determined by spectroscopy at 260 nm.

The clade B HIV-1 panel (NIH AIDS Reagent and Reference Program) [16], consists of virus clones SVPB11 (PVO, clone4), SVPB12 (TRO, clone 11), SVPB13 (AC10.0, clone29), SVPB14 (pRHPA4259, clone 7), SVPB15 (pTHRO4156, clone 18), and SVPB16 (pREJO4541, clone 67).

Two days prior to transfection, 2.6×10^6^ 293T cells were seeded in a 10 cm corning plate, using either complete DMEM (1% Penicillin/Streptomycin and 7.5% FBS) or in medium lacking antibiotics. Transfection was performed using Lipofectamine (Invitrogen) diluted 1∶3 in serum free medium. This was followed by the addition of the rev/env clone (12 µg) and the HIV-1 env-deficient backbone (24 µg). After a 20 min incubation at 37°C and 5% CO_2_, the mixture was added to the 293T cells, followed by another incubation period of 48 h. In order to provide necessary growth conditions, 15 ml of serum free medium was added to the cells at 4 hours post transfection. The virus was then harvested and collected using a 0.45 µm syringe filter and stored at −80°C.

### Virus Titration

Titrations were performed to determine the amount of virus produced during the transfection. This assay was done using TZM-bl cells, which are derived from HeLa cells and engineered to express HIV receptors such as CD4 and CCR5 [17]. These cells also contain the luciferase and beta-galactosidase genes under the control of the HIV-1 long terminal repeat [18]. Serial dilutions of virus were prepared in 96 well plates using medium containing 75 µg/ml of DEAE-dextran. Previously prepared TZM-bl cells were added to each well for a final concentration of 200 cells/ml. The titration plate was then maintained for 48 h at 37°C and 5% CO_2_. Infection of cells was measured indirectly using a Mammalian β-galactosidase assay kit (PIERCE) followed by an absorbance reading at 415λ. An absorbance of greater than 0.2, was considered a positive infection. The Tissue Culture Infectious Dose (TCID50) was determined for each viral stock per 1 ml by identifying the dilution of virus in which 50% of the TZM-bl cells were infected, as measured by the presence of β-galactosidase. It was determined that doses of 200 TCID50 were sufficient for experimentation and the virus was stored at −80°C.

### Virus blocking experiments

The Cc-CTRL (no insert), Cc-CD4, and Cc-MIP1a Caulobacter constructs were grown and prepared one day prior to an experiment. Experiments were carried out in triplicate wells of 96 well plates (at 3 identical wells per experimental condition) using Caulobacter at a concentration of 10^8^, 10^7^, and 10^6^ cells/ml. The TZM-bl cells were prepared in a manner as previously described. The volume of virus added was determined by previously calculating the 200TCID_50_ value. The virus was incubated for 1 h with the CD4 construct before the addition of TZM-bl cells. This allows sufficient binding of the bacteria to its predicted targets. In contrast, the MIP1a and control constructs were first exposed to TZM-bl cells for 1 h prior to virus addition. It was also necessary to determine the percent of infectivity when two of the constructs were combined (Cc-CD4 and Cc-MIP1a) and in this instance, both recombinant caulobacters were incubated to cells first and virus added 1 h later. After an overnight incubation at 37°C in 5% CO_2_, the plates were centrifuged at 800 rpm for 5 min and medium was changed in all wells. 48 h after initial incubation the viral titer was analyzed as previously described. As a positive control for inhibition of infection, the HIV gp120 specific monoclonal antibody, 2F5 was added to the assay of each experiment in triplicate. Data are presented and determined as a percentage of infection of the untreated control infection wells with the background from uninfected subtracted out.

### Statistical analysis

Statistical analysis was performed with Prism GraphPad software. The unpaired Student's t-test was used for statistical analysis. A P value of less than 0.05 was considered significant and values are reported in the respective legends.

## Results

### Expression of MIP1α and single domain CD4, independently within the S-layer protein of Caulobacter

**Figure 1:**
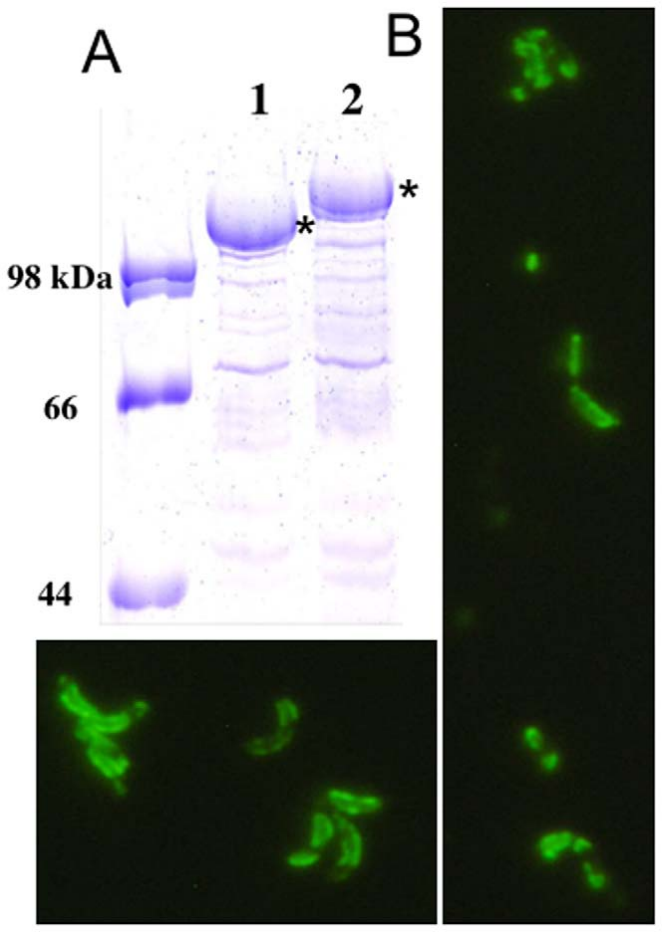
Display of CD4 domain 1 on Caulobacter. A. SDS-PAGE of normalized low pH extraction of S-layer protein (RsaA) from *C. crescentus* JS 4022. Lane 1- RsaA obtained from Cc-CTRL. Lane 2- RsaA obtained from Cc-CD4. Asterisks indicate the RsaA proteins. B. Fluorescence microscopy using anti-CD4 polyclonal antibody and an Alexa488-labeled secondary.

**Figure 2:**
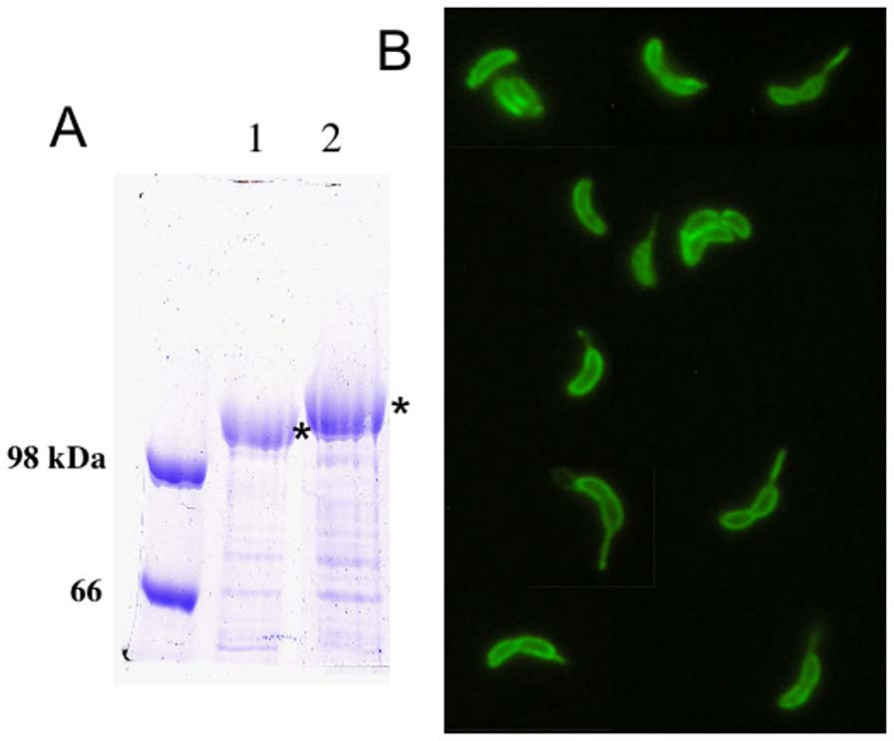
MIP1α surface display on Caulobacter. A. SDS-PAGE of normalized low pH extraction of S-layer protein (RsaA) from *C. crescentus* JS 4022. Lane 1- RsaA obtained from Cc-CTRL. Lane 2 - RsaA obtained from Cc-MIP1a. B. Fluorescence microscopy using anti-MIP1α polyclonal antibody and an FITC-labeled secondary.

To generate recombinant Caulobacter with expression of heterologous proteins within its surface S-layer protein, two separate vectors expressing either MIP1α or a single domain of CD4 were generated using p4ARsaA(723)/cmyc, an expression vector for *C. crescentus*, containing a version of *rsa*A (the S-layer gene) carrying restriction sites for insertion of genetic material at a site corresponding to amino acid 723 of the S-layer protein [11]. Using SDS-PAGE, we determined that both recombinant vectors (Cc-MIP1a, Cc-CD4) successfully generated chimeric S-layer proteins following transformation into *C. crescentus* (Fig. 1, 2). Further, immunofluorescent staining and microscopy of the recombinant Caulobacters with antibodies specific for MIP1α or CD4 demonstrated uniform staining across the bacterial cell surface, including the cell stalk, consistent with a complete coverage of the cell with the modified S-layer (Fig. 1, 2). Notably, immunostaining of *C. crescentus* expressing the single domain CD4 with the conformation dependent antibodies, SIM.2 and SIM.4, demonstrated proper conformation of the CD4 structure on the surface and this strongly suggested binding capability for gp120. In all cases, immunostaining of Caulobacters displaying an RsaA with no genetic insertion gave a negative result (not shown).

### Inhibition of HIV-1 infection with recombinant Caulobacter expressing either MIP1α or single domain CD4 with the S-layer

**Figure 3:**
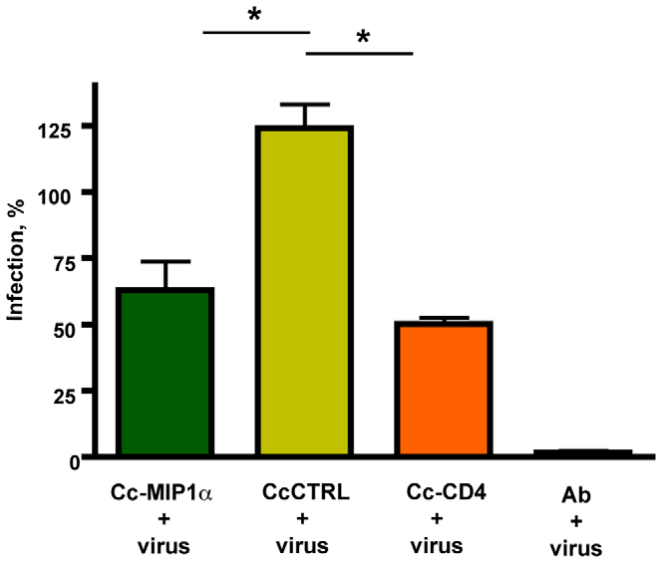
Surface expression of MIP1α or CD4 on Caulobacter is sufficient to inhibit infection with pseudotype HIV-1 subtype B virus clone SVPB13. The recombinant Caulobacters were co-incubated with HIV pseudotype virus SVPB13 and TZM-bl cells to demonstrate inhibition of infection. TZM-bl cells were also incubated alone, with virus, or with virus and neutralizing monoclonal antibody. Pseudotype virus infection was measured by ELISA for β-galactosidase. Significant levels of inhibition of infection were observed (denoted by asterisks) between both Cc-MIP1a and the Cc-CTRL (<0.001) and Cc-CD4 and Cc-CTRL (<0.01). HIV infections are presented as a percentage of the untreated control infections using the same pseudotype virus, SVPB13 and with the background for uninfected cells subtracted out. Each separate experiment was performed with 3 assay wells per condition. Data represent mean + standard error of the mean (s.e.m) from 4 separate experiments.

**Figure 4:**
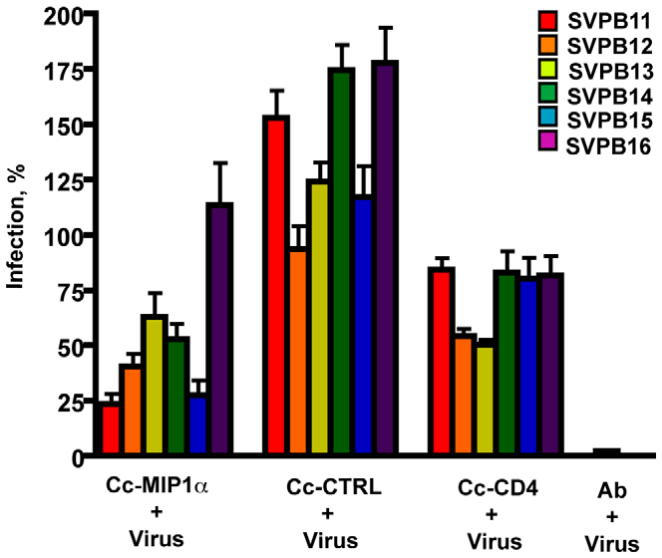
Surface expression of MIP1α or CD4 on Caulobacter is sufficient to inhibit infection with a number of pseudotype HIV-1 subtype B viruses. The recombinant Caulobacters were co-incubated with one of six different HIV pseudotype viruses and TZM-bl cells to demonstrate inhibition of infection. TZM-bl cells were also incubated alone, with virus, or with virus and neutralizing monoclonal antibody. Pseudotype virus infection was measured by ELISA for β-galactosidase. Significant levels of inhibition of infection were observed between both Cc-MIP1a and the Cc-CTRL (<0.001 for all the pseudotype clones) and Cc-CD4 and Cc-CTRL (<0.001 for SVPB11, SVPB13, SVPB14, SVPB16 and <0.01 for SVPB12, and SVPB15). HIV infections are presented as a percentage of the untreated control infections using the same pseudotype virus and with the background for uninfected cells subtracted out. Each separate experiment was performed with 3 assay wells per condition. Data represent mean + s.e.m from 3–4 separate experiments.

To determine whether expression of either MIP1α or single domain CD4 was sufficient to block infection of HIV-1, the recombinant *C. crescentus* were co-incubated with pseudotyped HIV-1 and TZM-bl cells in standard single cycle infection assays. Our initial studies with a single member of the clade B reference strain SVPB13, showed significant inhibition with both constructs (Figure 3) and warranted further study using the complete clade B reference panel from the NIH AIDS Research and Reference Reagent Program. We compared the ability of the two recombinant Caulobacters to inhibit different strains of HIV and better evaluate the potential general effectiveness of these specific reagents as potential microbicides (Figure 4).

Cc-MIP1a provided statistically significant protection from infection when compared to the Cc-CTRL (lacking MIP1α expression) (Figure 3, 4). Depending on the reference HIV-1 clone, the block in infection ranged from 35–78%. Co-incubation with Cc-CD4 also varied depending on the reference HIV-1 clone and effectively blocked from 22–56% of infection. Effective inhibition by Cc-MIP1a was not observed in 1 of the reference clones. The observed variability was likely due to the variation in gp120 sequence across the panel of HIV-1 clones as previously reported by Li et al. using both monoclonal antibody and soluble CD4 inhibitors [16].

### Comparing heat inactivated Caulobacter to recombinant Caulobacter

**Figure 5:**
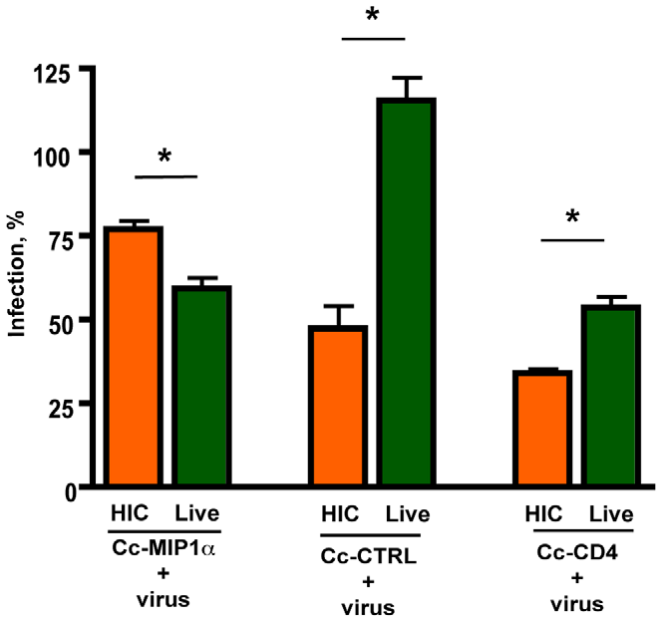
Heat inactivation of the recombinant Caulobacters retains inhibition of infection for pseudotype HIV-1 subtype B virus clone SVPB13. The recombinant Caulobacters were heat inactivated and co-incubated with HIV pseudotype virus SVPB13 and TZM-bl cells to demonstrate inhibition of infection. TZM-bl cells were also incubated alone, with virus, or with virus and neutralizing monoclonal antibody. Pseudotype virus infection was measured by ELISA for β-galactosidase. Significant levels of inhibition of infection were observed (denoted by asterisks) between HIC Cc-MIP1a and Live Cc-MIP1a (<0.005), HIC Cc-CTRL and Live Cc-CTRL (<0.001), and HIC Cc-CD4 and Live Cc-CD4 (<0.001). HIV infections are presented as a percentage of the untreated control infections using the same pseudotype virus, SVPB13 and with the background for uninfected cells subtracted out. Each separate experiment was performed with 3 assay wells per condition. Data represent mean + s.e.m from 3 separate experiments.

Continued development of this approach as a microbicide agent will likely involve the use of inactivated bacteria. Although inhibition had been demonstrated with co-incubation with live Caulobacter, the environmental conditions of the assay did not promote Caulobacter growth and survival. To preliminarily test the effectiveness of inactivated Caulobacter on inhibition of HIV-1 infection, the recombinant bacteria were heat inactivated (HIC) prior to co-incubation in the single cycle assays. Both Cc-MIP1a and CC-CD4 were found to effectively inhibit HIV-1 infection whether heat inactivated or not (Figure 5). Curiously, the control Caulobacter was found to be more effective at inhibiting infection following heat inactivation.

### Combinatorial effects on HIV-1 inhibition of co-incubation with Cc-MIP1a and Cc-CD4

**Figure 6:**
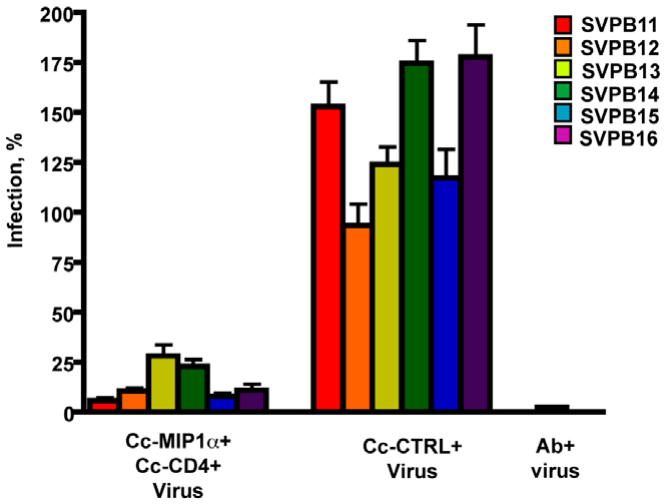
Incubation of both recombinant Caulobacters with pseudotype HIV-1 shows combinatorial effects and heightened inhibition of infection against the clade B viruses. The recombinant Caulobacters were combined in equal amounts and co-incubated with one of six different HIV pseudotype viruses and TZM-bl cells to demonstrate inhibition of infection. TZM-bl cells were also incubated alone, with virus, or with virus and neutralizing monoclonal antibody. Pseudotype virus infection was measured by ELISA for β-galactosidase. Significant levels of inhibition of infection were observed between both Cc-MIP1a/Cc-CD4 and the Cc-CTRL (<0.01 for all six pseudotype viruses). HIV infections are presented as a percentage of the untreated control infections using the same pseudotype virus and with the background for uninfected cells subtracted out. Each separate experiment was performed with 3 assay wells per condition. Data represent mean + s.e.m from 3–4 separate experiments.

It is likely that to achieve the broadest and most effective inhibition of HIV-1 infection a cocktail of several recombinant Caulobacters expressing different infection blockers will be required. To test the effectiveness of a cocktail strategy, co-incubation of Cc-MIP1a and Cc-CD4 with the pseudotyped HIV-1 and TZM-bl cells in single infection assays was performed. Co-incubation with both recombinant reagents demonstrated statistically significant enhanced blocking abilities against all reference strains tested, ranging from 74–98% inhibition of infection (Figure 6). Interestingly, against specific clade representatives like SVPB16, the individual recombinant Caulobacters poorly inhibited, yet in combination infection was reduced more than 80%. In no case was the combination of constructs observed to inhibit less than that observed for the individual recombinants. This observed enhancement validates the view that an effective microbicide would consist of multiple combinations of specific binding to maximally inhibit HIV infection.

## Discussion

The primary question posed in this study was whether the innocuous bacterium *C. crescentus* and its ability to display relatively large protein segments could be used to devise HIV specific blocking agents by mimicking the ligands involved with the interaction of the virus with its host cell. This minimally required success in achieving secretion of the chimeric S-layer protein, dense surface display and correct folding of the displayed segments. This was achieved and we were able to block infection in the standard TZM-bl assay for lentiviral infection by either blocking the host cell co-receptor (via MIP1α display) or the virus itself, via CD4 display. Each performed this function separately, reducing infectivity by about half and when the two constructs were combined into one assay infection inhibition worked in an additive fashion, achieving nearly complete blockage.

It is reasonable to presume that correct or nearly correct folding occurred for both MIP1α and CD4 when displayed on Caulobacter, given the ability to bind specific antibodies and the infection blocking activity noted. It is also highly likely that the high level expression and the S-layer mediated display of the ligands compensated for any less than perfect folding by providing multiple binding opportunities. Normal levels of Caulobacter S-layer protein are unusually high for a bacterium (approximately 25% of total cell protein (manuscript submitted)). In this instance we calculate that even the displayed recombinant portion alone accounts for 1–2% of total cell protein and all of this is displayed on the outermost surface of the bacterium. Thus, we expect that the number of cells required to achieve a practical level of inhibition of infection is likely significantly less than the lower level display of CD4 reportedly observed on other bacteria such as Lactobacilli [6], [7], [8], [19].

Variation in inhibition was observed across the different HIV-1 reference strains. Notably, the greatest variability was found against the MIP1α construct and this is likely due to the high degree of variability across gp120 for the individual pseudoviruses. Prior work by Li et al. [16] demonstrated that across these 6 pseudovirus and a number of others that a significant level of sequence variation exists and this variation is directly responsible for differences in sensitivity and resistance of these viruses to env-based neutralization. Prior to MIP1α binding to CCR5, proper engagement of CD4 and gp120 followed by a folding event is required to expose the full binding site. This complex binding likely challenges the specific interactions of MIP1α with HIV env. To develop the most effective measure, it is likely that a cocktail of multiple recombinant Caulobacters expressing different HIV-1 blocking agents will inhibit the widest range of potential HIV-1 variants. This was clearly effective in our system as combinations of both recombinant Caulobacters resulted in nearly complete inhibition of infection.

The development of a bacteria based microbicide has an expectation that the supply of cells is relatively inexpensive to produce. Caulobacters are in fact simple to grow and can be readily fermented to high densities in shake flasks or standard fermenters in media containing only glucose, salts and ammonia as a nitrogen source. As such, the development of Caulobacter as a microbicide platform is a sound low cost choice in terms of productivity. An important issue is whether Caulobacter is safe for exposure to mucosal tissues. This has not yet been tested directly, but the low endotoxin LPS [9] and the record of no septic or other overt symptoms with intraperitoneal injections in mouse model systems [20] suggests that it will be and that experiments to determine safety are warranted.

The presumption in the use of engineered Caulobacters as a microbicide is that it would not be used as a commensal bacterium but rather as an agent that would be applied as a topical agent prior to any perceived exposure or risk for infection (typically through sexual acts or the process of childbirth with an HIV positive mother). Caulobacters are not normal inhabitants of humans and will not grow in this environment. As such, they are merely a platform for the generation and presentation of the blocking agent. Thus, this obviates any immediate concerns that would be common to the use of a commensal bacterium for this purpose - that is, being able to compete with normal flora or alternatively, if it can compete, that long-term maintenance may result in unwanted effects that are then difficult to reverse. Further, it is our expectation that killed Caulobacters will be used. Ultimately, this will be performed via heat, irradiation or chemicals such as formalin or beta-propriolactone. In preliminary studies with heat killed cells, blocking activity was relatively similar to that observed for live cells, except that heat inactivated control cells had an unexpectedly high level of inherent blocking activity. This non-specific effect may be helpful (additive) to the specific blocking ligands, or may be an indication that other killing methods will be more appropriate and is currently under investigation.

The exceptional ability of Caulobacter to tolerate the insertion of a range of heterologous peptides and proteins in to its S-layer structure has been demonstrated here and previously [2], [11], [21]. The ability of the native binding sites to remain intact following display was further described. Our future work is not limited to single constructs, as simultaneously expression of two or more modified S-layers is readily possible [22]. When considering pairs of displayed proteins, some are predicted to be potentially synergistic with current two activities and other agents, such as single chain antibodies directed to gp120 (for pairing with CD4 display) or ligands or antibodies directed to other T-cell or mucosal cell receptors for pairing with MIP1α. The ultimate goal is to produce the best combination of single and co-displayed ligands to maximize HIV infection inhibition and to minimize the opportunity to select for HIV isolates that evade the blocking activity by mutation. An additional benefit in evaluating combinations of ligands may be in discovering unexpected synergistic infection inhibition, such as was noted with SVPB16.
